# Prognosis‐oriented molecular subtypes of retroperitoneal liposarcoma

**DOI:** 10.1002/ctm2.70050

**Published:** 2024-10-15

**Authors:** Mengmeng Xiao, Da Qin, Xiangji Li, Fanqin Bu, Shixiang Ma, Xiaobing Chen, Yu Zhao, Chenghua Luo, Li Min

**Affiliations:** ^1^ Department of General Surgery Peking University People's Hospital Beijing P. R. China; ^2^ Department of Gastroenterology State Key Laboratory for Digestive Health National Clinical Research Center for Digestive Disease Beijing Digestive Disease Center Beijing Key Laboratory for Precancerous Lesion of Digestive Disease Beijing Friendship Hospital Capital Medical University Beijing P. R. China; ^3^ Department of Retroperitoneal Tumor Surgery Peking University International Hospital Beijing P. R. China

Dear Editor,

Retroperitoneal liposarcoma (RPLS) is an extremely rare malignant tumour.^1^ It is largely understudied with unknown risk factors and limited treatment options. The rapid development of next‐generation sequencing technology has brought the diagnosis and treatment of neoplastic diseases to the era of precision medicine.^2^ It provided detailed RNA‐seq information for the prognosis and prediction of different therapies and guided the crucial clinical decision‐making processes throughout the treatment.^3^ Here, we aim to develop prognosis‐oriented molecular subtyping of RPLS and further provide novel treatment strategies for RPLS patients (Figure 1A). This work fundamentally differs from our previous reports.^4^ It focuses on prognostic genes rather than differential genes, employing Weighted Gene Network Analysis and nonnegative matrix factorization (NMF) algorithms to achieve a more refined molecular classification of RPLS. By selecting and validating representative molecular biomarkers for each subtype, we have further streamlined the classification system for clinical application. Additionally, this classification system elucidates how the molecular biological characteristics of the different subtypes influence distinct clinical prognoses.

**FIGURE 1:**
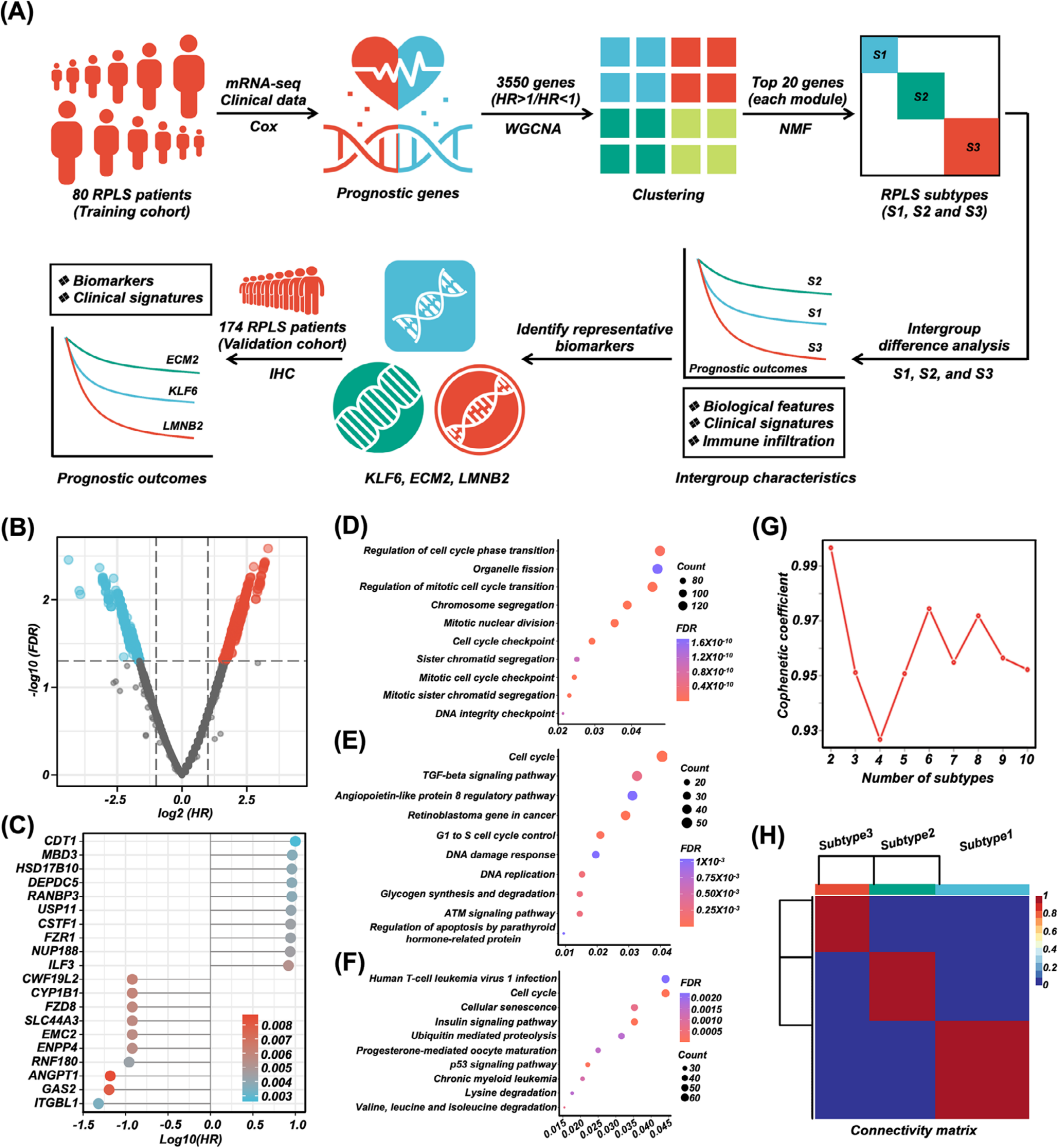
Functional enrichment and WGCNA clustering of RPLS prognostic genes. (A) Schematic overview of the study design. (B) Volcano plot depiction of prognostic genes associated with OS in the training cohort. (C) Lollipop plot of the top 20 prognostic genes associated with OS in the training cohort (HR > 1 and HR < 1). (D–F) Bubble plots of prognostic genes in GO (D), enrichWP (E), and KEGG (F). (G, H) NMF clustering by top 20 prognostic genes of WGCNA modules.

Research Registration Unique Identifying Number (UIN)

1. Name of the registry: ClinicalTrials.gov
2. Unique identifying number or registration ID: NCT03838718
3. Hyperlink to your specific registration: https://clinicaltrials.gov
4. The sequencing data have been deposited at the Open Archive for Miscellaneous Data (OMIX) database of the China National Center for Bioinformation (CNCB) under the accession number OMIX002786

To construct prognosis‐oriented RPLS molecular subtypes, we first identify the prognostic genes in our training cohort based on univariate cox regression (*N* = 80, Table 1; Tables S1 and S2). The regression analysis pipeline was conducted with the R package “survival” (Supporting Information Material and methods). A total of 3550 genes were associated with OS and DFS (*p *< .05; Figure 1B), and the top 10 genes of HR > 1 and HR < 1 were shown in Figure 1C. Functional annotation revealed these genes were enriched in the cell cycle, TGFβ‐signalling pathway, angiopoietin, and cellular senescence (Figure 1D–F). We then functionally clustered 3550 prognostic genes by WGCNA (Figure S1) and selected the top 20 genes in each module for NMF grouping. Three RPLS subtypes were significantly distinguished (Figure 1G–H). Log‐rank analysis showed that subtype 2 (S2) had the best prognostic outcomes (including OS, *p* < .0046; DFS, *p* < .0001) compared with subtype 1 (S1) and subtype 3 (S3), while S3 had the worst prognostic outcomes among them (Figure 2A,B). Functional annotation of characteristic genes showed that “Obesity”, “Overnutrition”, and “*PPAR* signaling pathway” were mainly enrichment terms of S2 (Figure S2).

**TABLE 1:** Baseline characteristics of training cohort and validation cohort.

	Training cohort (*N* = 80)	Validation cohort (*N* = 174)	*P*‐value
Age (years)	56.5 (28–80)^a^	56 (29–83)^a^	0.654
Sex			
Male	37 (46.25)	87 (50.00)	0.579
Female	43 (53.75)	87 (50.00)	
Pathology			
WDLS	29 (36.25)	53 (30.36)	0.392
DDLS	48 (60.00)	108 (62.07)	
MLS and PLS	3 (3.75)	8 (4.60)	
NR	0 (0)	5 (2.97)	
Surgery times^b^			
0–1	50 (62.50)	106 (60.92)	0.810
2–7	30 (37.50)	68 (39.08)	
Tumour size			
All	17.70 (3–39.90)^a^	16.70 (2.7–39.90)^a^	0.172
20 cm	45 (56.25)	107 (61.49)	0.119
≥20 cm	34 (42.50)	57 (32.76)	
NR	1 (1.25)	10 (5.75)	
MDM2 score		MDM2 (amplification)	
≤2	62 (77.50)	Yes: 130 (74.71)	NA
>2	14 (17.50)	No: 26 (14.94)	
NR	4 (5.00)	NR: 18 (10.35)	
KLF6 score	NA	1.20 (0.1–2.85)^a^	NA
KLF6 strength			
1	NA	53 (30.46)	NA
2	NA	67 (38.51)
3	NA	54 (31.03)	
ECM2 score	NA	1.10 (0.1–2.85)^a^	NA
ECM2 strength			
1	NA	64 (36.78)	NA
2	NA	52 (29.89)	
3	NA	58 (33.33)	
LMNB2 score	NA	1.80 (0.1–2.85)^a^	NA
LMNB2 strength	NA		
1	NA	20 (11.50)	NA
2	NA	62 (35.63)	
3	NA	92 (52.87)	

^a^ The data is shown as Median (minimum, maximum); other data are shown as Number (%).

^b^ The definition of surgical times is the sum of current admission surgery and previous surgical resection.

Abbreviations: DDLS, dedifferentiated liposarcoma; ECM2, extracellular matrix protein 2; KLF6, KLF transcription factor 6; LMNB2, Lamin B2; MDM2, mouse double minute 2; MLS, myxoid liposarcoma; NA, not applicable; NR, not reported; PLS, pleomorphic liposarcoma; WDLS, well‐differentiated liposarcoma.

**FIGURE 2:**
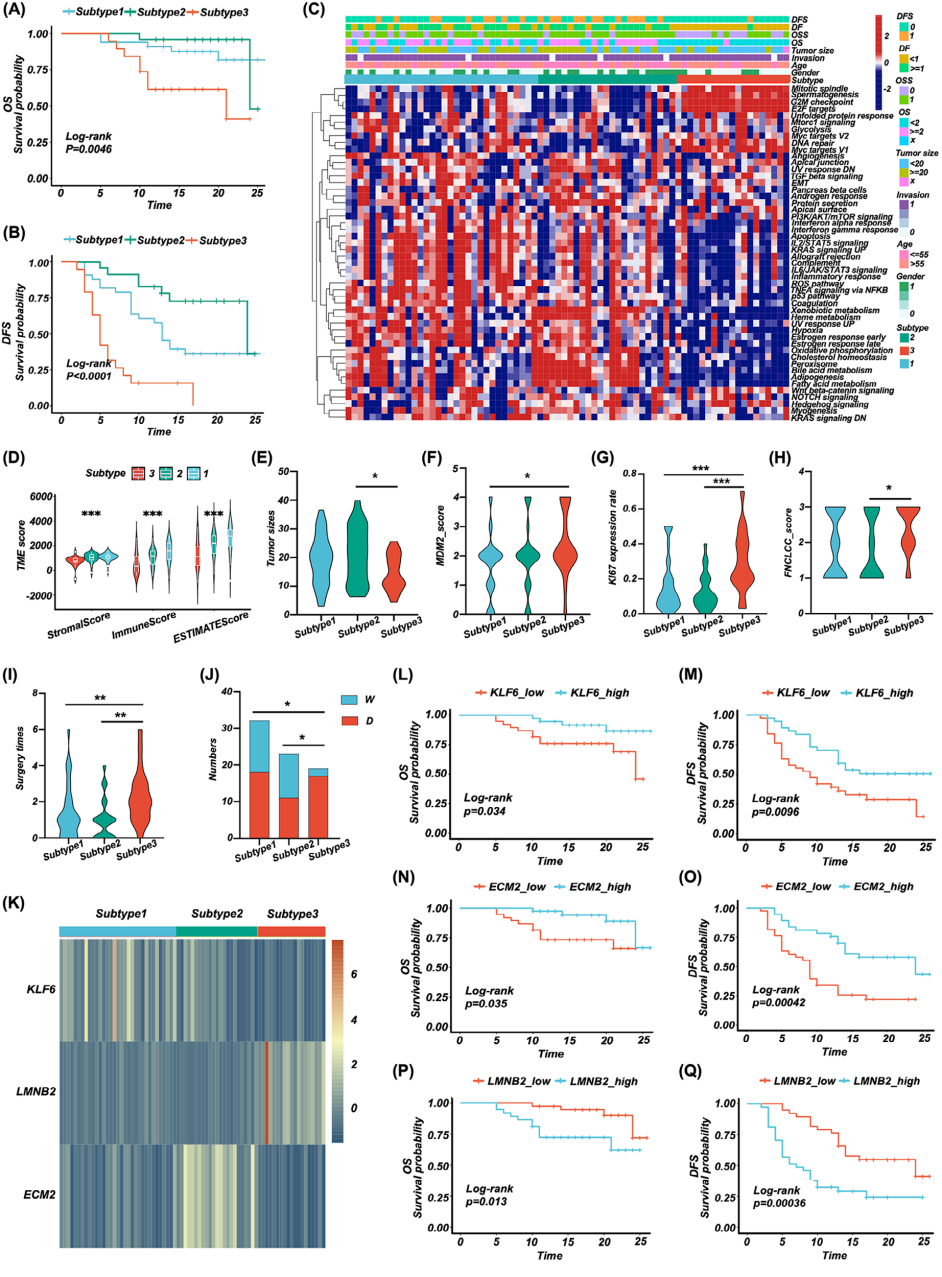
Prognosis‐oriented molecular subtypes of RPLS and representative biomarkers of RPLS subtypes. (A, B) Log‐rank survival curves of OS (A) and DFS (B) in different subtypes. (C) The hierarchical heatmap of hallmark gene sets in different subtypes. (D) Violinplots of the tumour microenvironmental scores in different subtypes. (E–I) Violinplots of tumour size (E), MDM2 score (F), Ki67 expression (G), FNCLCC score (H), and surgery times (I) in different subtypes. (J) Bar plot of the composition ratio of WDLS and DDLS in different subtypes. (K) Heatmap of the representative biomarkers (KLF6/S1, ECM2/S2, and LMNB2/S3) in different subtypes. (L, M) Log‐rank survival curves of OS (L) and DFS (M) in KLF6‐low and ‐high expressed groups. (N, O) Log‐rank survival curves of OS (N) and DFS (O) in ECM2‐low and ‐high expressed groups. (P, Q) Log‐rank survival curves of OS (P) and DFS (Q) in LMNB2‐low and ‐high expressed groups.

Hallmark gene set of cancer well represents the specific biological processes and states of tumours. We previously revealed the differences in the functional annotation among subtypes. To verify them, hallmark gene sets were scored on patients of each subtype by ssGSEA. We found that metabolism‐related pathways, including “adipogenesis” and “bile acid metabolism”, were most strongly associated with S2. This observation is consistent with recent studies that elucidate the role of bile acids in modulating the tumour immune microenvironment, as well as their involvement in lipogenesis, metabolism, and the regulation of tumour proliferation and apoptosis.^5^, ^6^, ^7^ These findings underscore the critical role of bile acids in tumorigenesis and progression. Proliferation‐related pathways, such as “mitotic spindle” and “G2M checkpoint” were the most important biological processes in S3, while immune‐related pathways, such as “TNFα”, “IL2‐STAT5” and “IFNα and IFNβ response” were mainly enriched in S1 (Figure 2C). Although activation of immune responses can significantly inhibit tumour progression, concomitant upregulation of PI3K‐Akt‐mTOR and KRAS signalling pathways reduces S1 prognosis.^8^ We also evaluated the tumour microenvironment (TME, using the ESTIMATE algorithm) and clinical signatures (using one‐way ANOVA) among three subtypes (Supplementary material and methods), the results showed that S3 had the lowest TME score and smallest tumour size (Figure 2D,E), but had the highest MDM2 expression, Ki67 index and FNCLCC score (Figure 2F,H), the most surgery times (Figure 2I), and the most significant pathological subtype ratio (DDLS/WDLS) among them (Figure 2J). These results demonstrated that the subtypes exhibited distinct biological features connecting the clinical, pathological, and prognostic signatures of RPLS.

For the NMF classification of RPLS patients, KLF6, ECM2, and LMNB2 were identified as representative biomarkers of S1, S2, and S3, respectively (Figure 2K and Figure S3). We then performed log‐rank analysis according to the expression level of the three biomarkers, and the results showed that RPLS patients with high expressed KLF6 and ECM2 had better OS (KLF6: *p* = .034; ECM2: *p* = .035; Figure 2L,N) and DFS (KLF6: *p* = .0096; ECM2: *p* < .001; Figure 2M,O). However, Patients with high expressed LMNB2 had worse OS (*p* = .013, Figure 2P) and DFS (*p* < .001, Figure 2Q). KLF6 and ECM2 were important tumour suppressors in many tumours, they acted a beneficial role by inactivating p38/JNK/ERK signalling and increasing p21 in a p53‐independent manner.^9^, ^10^ LMNB2 is crucial in maintaining the integrity of the nuclear skeleton and participating in cell proliferation, ageing, and DNA damage repair.^11^ It promotes the progression of these tumours by silencing p21, ki67, and caspase3, activating CDCA3, and regulating immune infiltrates.^12^, ^13^, ^14^ These results suggest that the representative biomarkers are prognostic molecules in nature, which echoes our initial design idea of establishing prognosis‐oriented molecular subtypes of RPLS.

Finally, we validated the representative biomarkers in the Retroperitoneal Sarcoma Registry (RESAR) cohort (*N* = 174, NCT03838718, Table 1). IHC staining was performed as previously described (Supporting Information Material and Methods) and the representative images with different degrees of expression are shown in Figure 3A. These classifications are derived from a comprehensive evaluation of biomarkers’ expression across all validation cohort samples, with the lowest one‐third designated as low expression, the middle one‐third as intermediate expression, and the highest one‐third as high expression. We found that RPLS patients with higher *KLF6* and *ECM2* had better OS (Figure 3B,D) and DFS (Figure 3C,E) than those with lower expression, whereas patients with higher *LMNB2* had worse OS and DFS than patients with lower expression (Figure 3F,G). It is consistent with our previous findings in our training cohort. To further validate the three‐gene‐based molecular subtypes, we divided RPLS patients into three subgroups for prognosis analysis according to which biomarker (*KLF6*, *ECM2*, and *LMNB2*) showed the highest expression (Table S3). The *ECM2* subgroup had the best OS and DFS, whereas the *LMNB2* subgroup had the poorest OS and DFS (Figure 3H,I). Besides, the highest pathological subtype ratio (DDLS/WDLS), *Ki67* level, and most surgery times (Figure 3J–L) were observed in the *LMNB2* subgroup, in accordance with disease progression.

**FIGURE 3:**
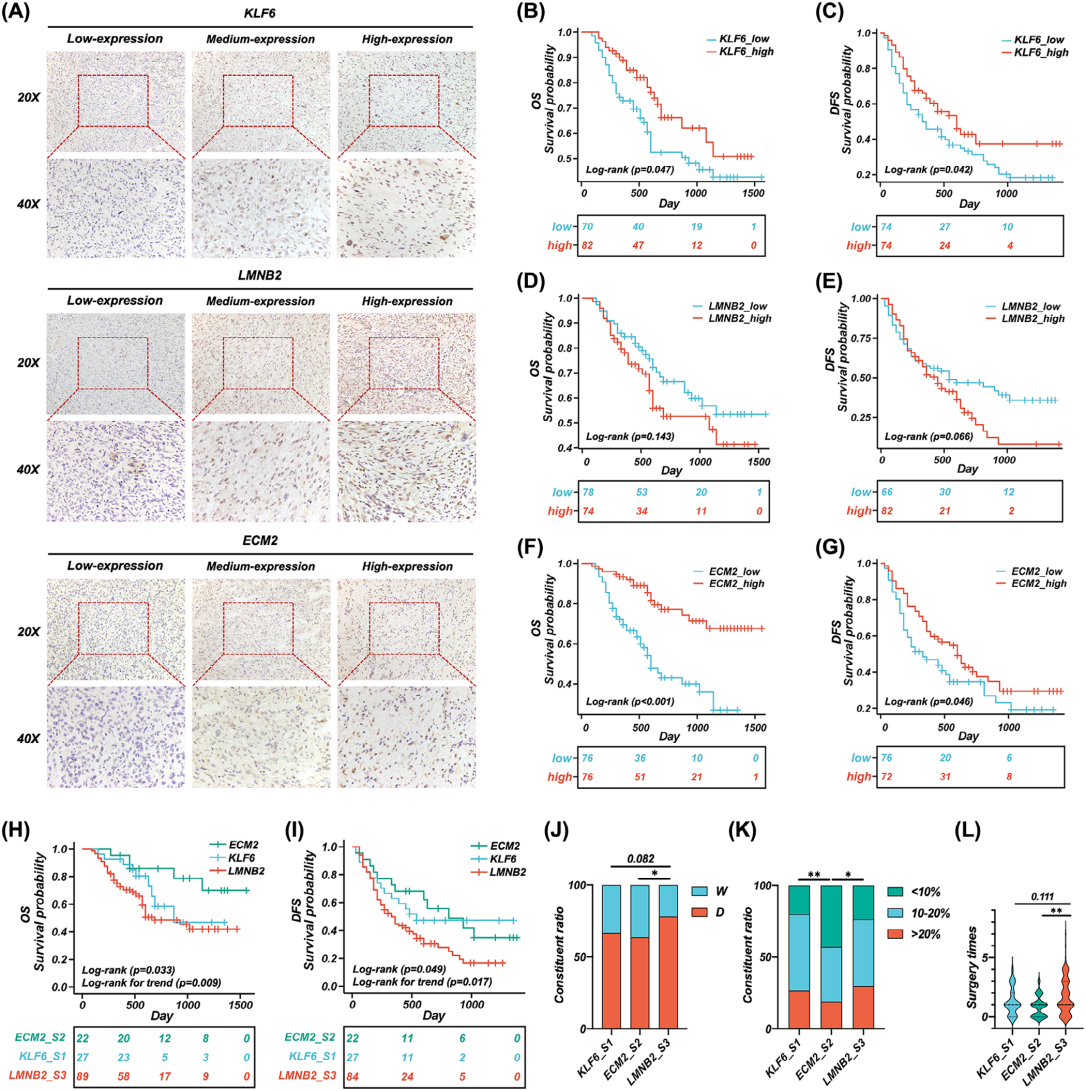
Validation of representative biomarkers and RPLS molecular subtypes in the RESAR cohort. (A) Representative IHC staining images of KLF6, LMNB2, and ECM2. (B, C) Log‐rank survival curves of OS (B) and DFS (C) in KLF6‐low and KLF6‐high expressed groups. (D, E) Log‐rank survival curves of OS (D) and DFS (E) in ECM2‐low and ECM2‐high expressed groups. (F, G) Log‐rank survival curves of OS (F) and DFS (G) in LMNB2‐low and ‐high expressed groups. (H, I) Survival curves of OS (H) and DFS (I) in KLF6‐, ECM2‐, and LMNB2 subtypes. (J, K) Bar plots of the composition ratio of WDLS and DDLS (J) and Ki67 level (K) in KLF6‐, ECM2‐, and LMNB2‐subtypes. (L) Violin plot of the surgery times in KLF6‐, ECM2‐, and LMNB2 subtypes.

To sum up, we proposed three RPLS subtypes with IHC‐verified biomarkers. The new classification of RPLS revealed distinct biological features and indicated various prognoses. It would be a preliminary but essential effort to bring RPLS treatment to the precision medicine era.

## AUTHOR CONTRIBUTIONS

Conception/design: Li Min, Chenghua Luo; provision of study material or patients: Chenghua Luo, Mengmeng Xiao, Shixiang Ma, Xiaobing Chen; collection and/or assembly of data: Da Qin, Xiangji Li, Fanqin Bu, Yu Zhao; data analysis and interpretation: Xiangji Li, Da Qin, Fanqin Bu; manuscript writing: Mengmeng Xiao, Xiangji Li, Fanqin Bu, Li Min; final approval of manuscript: Mengmeng Xiao, Da Qin, Xiangji Li, Fanqin Bu, Shixiang Ma, Xiaobing Chen, Yu Zhao, Chenghua Luo, Li Min.

## CONFLICT OF INTEREST STATEMENT

The authors declare no conflict of interest.

## FUNDING INFORMATION

This work was supported by grants from the Young Elite Scientists Sponsorship Program (2023QNRC001) and the National Natural Science Foundation of China (82073390). The study sponsors had no role in the design and preparation of this manuscript.

## ETHICS STATEMENT

Specimens of RPLS patients were obtained from Peking University International Hospital. The experiments were undertaken with the understanding and written consent of each subject. The study protocol conformed to the standards set by the Declaration of Helsinki and was approved by the Ethics Committee of Peking University International Hospital, Peking University Health Science Center (WA2020RW29).

## CONSENT FOR PUBLICATION

All authors have read and approved the manuscript and agree with submission to Clinical and Translational Medicine.

## Supporting information



Supporting information

Supporting information

Supporting information

Supporting information

Supporting information

## Data Availability

The datasets used and/or analyzed during this study are included in this letter or the supporting information files.
