# Supplementary material for: Prognosis‐oriented molecular subtypes of retroperitoneal liposarcoma
Source: Clin Transl Med. 2024 Oct 15;14(10):e70050. doi: 10.1002/ctm2.70050 (PMC11479751; doi:10.1002/ctm2.70050)
Supplement: Supplementary file 1 — Supporting information [file CTM2-14-e70050-s004.pdf]

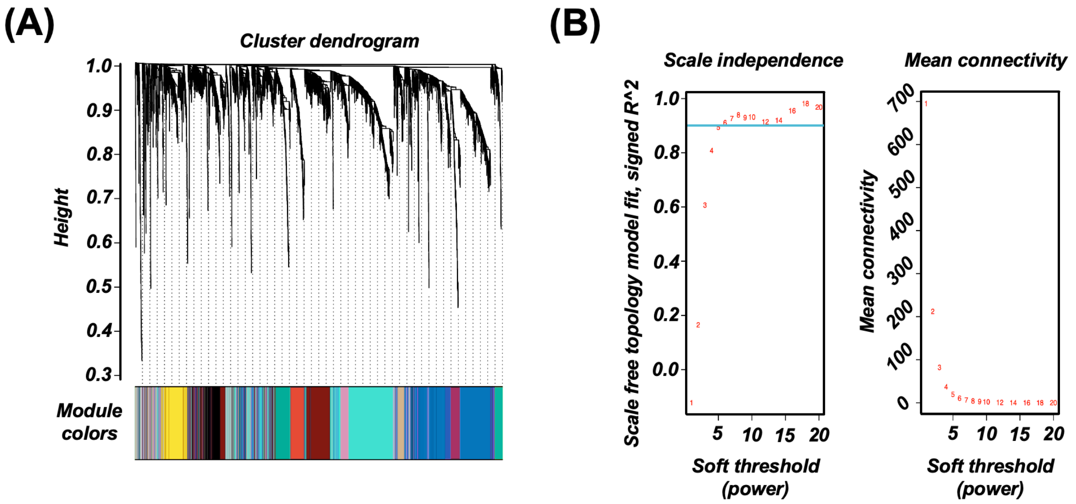


***Figure S1.*** Gene coexpression module construction of 3550 prognostic genes identified in training cohort.

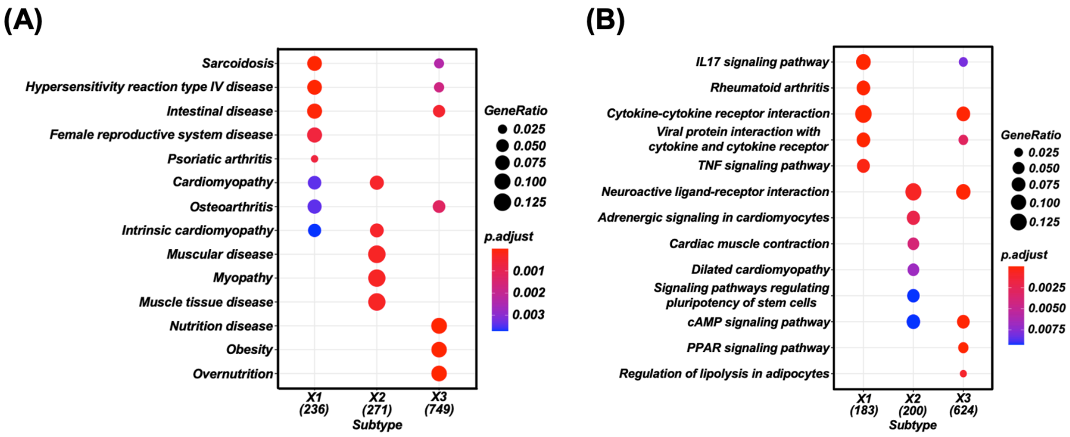


***Figure S2.*** Bubble plot of characteristic genes of different RPLS subtypes in GO (A) and KEGG (B).

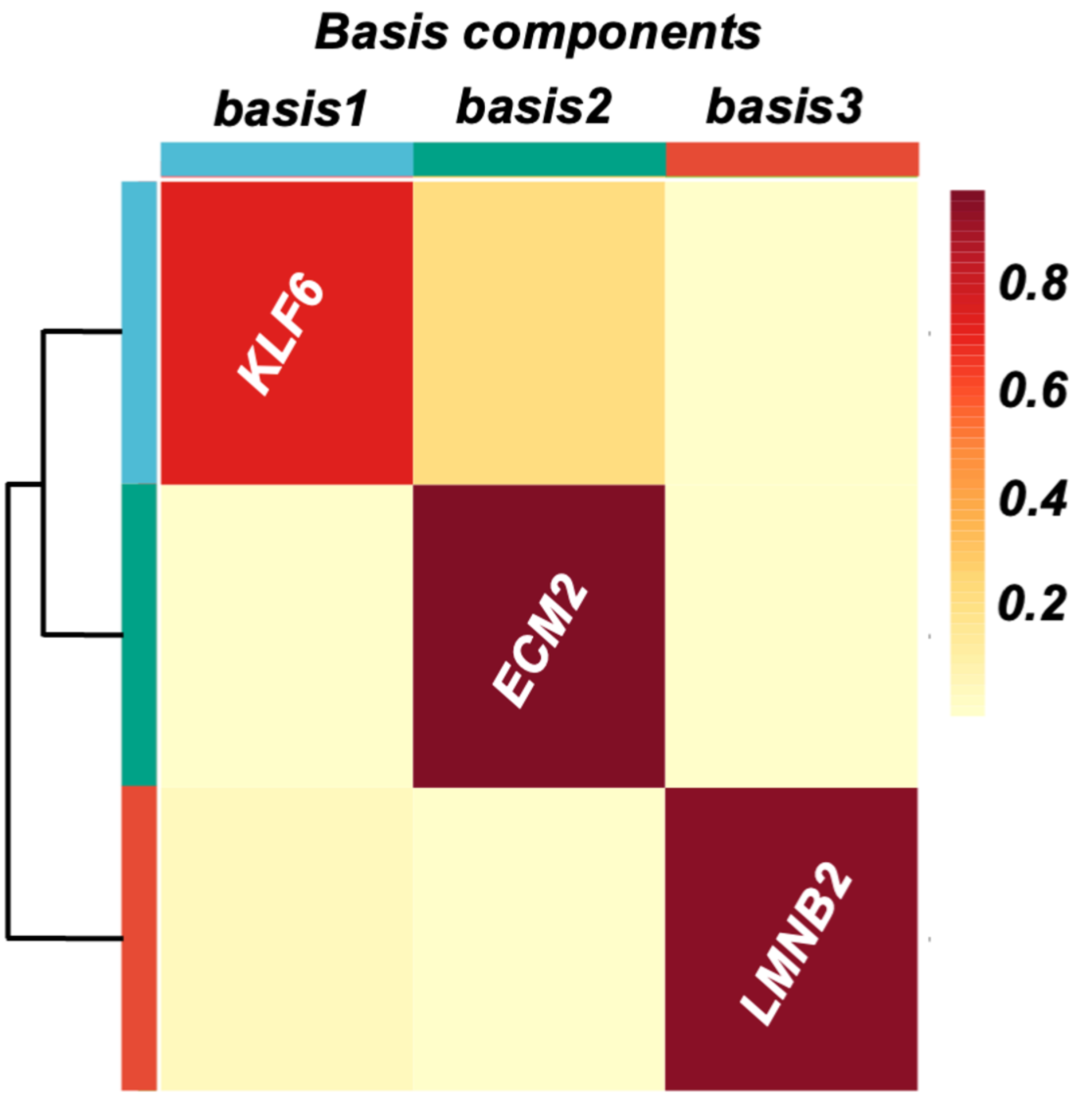


***Figure S3.*** Heatmap of the representative biomarkers (KLF6/S1, ECM2/S2, and LMNB2/S3) in different RPLS subtypes.
