# Supplementary material for: Prognosis‐oriented molecular subtypes of retroperitoneal liposarcoma
Source: Clin Transl Med. 2024 Oct 15;14(10):e70050. doi: 10.1002/ctm2.70050 (PMC11479751; doi:10.1002/ctm2.70050)
Supplement: Supplementary file 5 — Supporting information [file CTM2-14-e70050-s003.docx]

**SUPPLYMENTARY MATERIALS AND METHODS**

***Patients and tissue specimens***

Tumor specimens from a cohort of 80 RPLS patients (Training cohort, Table S1) and another cohort of 174 RPLS patients (Validation cohort, Table S3) were obtained from the Peking University International Hospital. Two cohorts are sourced from Retroperitoneal Sarcoma Registry (RESAR, NCT03838718). The specimens were snap-frozen in liquid nitrogen within 1 h and then stored in a -80 ℃ refrigerator before use. Clinical information such as surgery times, tumor sizes, and clinicopathological subtypes was collected from the medical records. No patients had undergone previous chemotherapy or radiation treatment. Overall survival (OS) was defined as the interval between the latest surgery and death from tumors or between the latest surgery and the last observation taken for surviving patients. Disease-free survival (DFS) was defined as the interval between the latest surgery and diagnosis of relapse or death. The experiments were carried out with the understanding and written consent of all the participants. The study protocol conformed to the standards set by the Declaration of Helsinki and was approved by the Ethics Committee of Peking University International Hospital, Peking University Health Science Center (WA2020RW29).

***Transcriptomics analysis***

The transcriptomics data were generated with five steps: sample preparation, library construction, DNA amplification, high throughput sequencing, and data analysis.

Total RNA was extracted from 80 snap-frozen tumor specimens (Training cohort, Table S1) using TRIzol Reagent (Invitrogen). RNA degradation and contamination were monitored with 1% agarose gel. RNA purity was checked by the NanoPhotometer spectrophotometer (IMPLEN, Los Angeles, CA, USA). RNA concentration was measured using the Qubit RNA Assay Kit with the Qubit 2.0 Fluorometer (Life Technologies, CA, USA). RNA integrity was assessed using the RNA Nano 6000 Assay Kit of the Agilent Bioanalyzer 2100 System (Agilent Technologies, CA, USA).

A total amount of 3-5 ug RNA per sample was used as input material for the RNA library. Sequencing libraries were generated using NEBNext® Multiplex Small RNA Library Prep Set for Illumina® (NEB, USA) following the manufacturer’s recommendations and index codes were added to attribute sequences to each sample. The clustering of the index-coded samples was performed on a cBot Cluster Generation System using TruSeq SR Cluster Kit v3-cBot-HS (Illumia) according to the manufacturer’s instructions. After cluster generation, the library preparations (Strand-specific cDNA) were sequenced on an Illumina NovaSeq 6000 platform, and single-end reads were generated (Novogene Bioinformatic Technology, Beijing, China).

FPKMs of both coding genes and lncRNAs in each sample were calculated by Cuffdiff (v2.1.1). FPKMs were the sum of the FPKMs of transcripts in each gene group and were calculated based on the length of the fragments and read counts mapped to this fragment (Table S2). These sequencing data have been deposited at the Open Archive for Miscellaneous Data (OMIX) database of China National Center for Bioinformation (CNCB) under the accession number OMIX002786.

***Identification of prognostic genes***

Univariate cox regression analysis was used to identify prognostic genes of RPLS. Both OS and DFS were calculated. An adjusted *p*-value<0.05 and |HR| value>1 was considered significant. The regression analysis pipeline was conducted with the R package “survival”.

***Gene functional annotation***

Functional enrichment was performed to elucidate the possible molecular pathways of the prognostic genes. We used the R package “clusterprofiler” to conduct gene ontology (GO) and Kyoto Encyclopedia of Gene and Genomes (KEGG) of prognostic genes, and the false discovery rate <0.05 was considered significantly enriched.

***Construction of weighted gene co-expression modules (WGCNA)***

To explore the interactions between genes, we performed WGCNA, a system biology tool that converts co-expression measures into connection weight or topology overlap measures. Genes involved in the same pathway or same functional compound were identified and clustered, and these genes tend to demonstrate a similar expression pattern. Both OS and DFS prognostic genes were inputted to construct weighted co-expression modules using the “WGCNA” R package. The threshold of the co-expression module was set as *p*<0.05.

***Consensus clustering with NMF***

NMF was used to identify molecular subtypes of RPLS. Specifically, NMF was applied to gene expression matrix *A* which contained the top 20 genes of each module of WGCNA. Matrix *A* was factorized into 2 nonnegative matrices *W* and *H*. Repeated factorization of matrix *A* was performed and its outputs were aggregated to obtain consensus clustering of RPLS samples. The optimal number of subtypes was selected according to cophenetic, dispersion, and silhouette coefficients. This consensus clustering was conducted with the R package “NMF”.

***Single-sample gene set enrichment analysis***

For the PKUIH-RPLS dataset, 50 hallmark signatures were first quantified for their enrichment degrees within respective RPLS samples using single-sample gene set enrichment analysis (ssGSEA) scores. The 50 hallmark gene sets summarize and represent specific well-defined biological states or processes and display coherent expression. The RPLS samples were hierarchically clustered into high, medium, or low groups based on ssGSEA scores.

In addition, the TME score was calculated using the ESTIMATE algorithm, a well-established bioinformatics tool that evaluates the immune and stromal components within the tumor microenvironment based on gene expression profiles. The ESTIMATE algorithm generates three key scores:

*(1) Immune Score:* This score reflects the presence of immune cell infiltrates in the tumor tissue, derived from the expression levels of genes associated with immune activity. A higher immune score indicates a more robust immune response.

*(2) Stromal Score:* This score assesses the contribution of stromal cells to the tumor microenvironment, indicating the degree of fibrotic tissue and other supportive elements present. A higher stromal score suggests a more pronounced structural support for the tumor.

*(3) ESTIMATE Score:* This is a composite score calculated by integrating the immune and stromal scores. It provides an overall assessment of the TME, with higher scores indicating a more complex and supportive microenvironment.

***Immunohistochemistry (IHC)***

The protocol was performed as previously described^1,2^. In brief, the KLF6, ECM2, and LMNB2 antibodies for IHC were purchased from Proteintech (Cat No: 10895-1-AP; 21376-1-AP; 14716-1-AP). With deparaffinization for 15min × 3 in dimethylbenzene and routine hydration, the tissues were soaked in phosphate buffer saline (PBS) for 10min and then performed high-pressure antigen retrieval (Tris-EDTA, PH=9.0) for 2.5min. After being treated with a 3% endogenous catalase blocker (ZSBIO, PV-6000) for 10min, the tissues were incubated in goat serum (ZSBIO, ZLI-9022) for the blocking of nonspecific reaction and then incubated with primary antibody (KLF6=1:20-200; ECM2=1:50-500; LMNB2=1:50-500) at 4℃ overnight. The next day, tissues were washed and incubated with goat anti-rabbit secondary antibody (ZSBIO, PV-9000) for 1h at room temperature, then washed and stained with DAB reagents (ZSBIO, ZLI-9018). Then hematoxylin staining, 1% hydrochloric acid alcohol differentiation, ammonia water anti-blue, and neutral gum sealing.

The IHC results were evaluated by pathologists, the staining extent was categorized as follows: 1 for 0-33%, 2 for 34-66%, and 3 for 67-100%. Additionally, the intensity of staining was scored based on expression levels, defined as weak staining (1), moderate staining (2), and strong staining (3). The final scores were calculated by the following formula:

$$Final score=Staining extent score\times Staining intensity score$$

The expression levels of KLF6, ECM2, and LMNB2 are stratified into three categories: low, medium, and high. These classifications are derived from a comprehensive IHC evaluation of biomarkers expression across all validation cohort samples, with the lowest one-third designated as low expression, the middle one-third as intermediate expression, and the highest one-third as high expression.

The correlation between KLF6, ECM2, and LMNB2 expression levels and the prognostic outcomes of RPLS patients was analyzed with Kaplan-Meier analysis.

***Statistical Analysis***

Continuous variables were summarized as mean±SD, categorized variables were described by frequency (N) and proportion (%). T-tests, nonparametric tests, One-way ANOVA, and chi-square tests were used in different variables. Prism 9.0 and R software (version 4.1.3) were conducted for statistical analyses. A difference of *p*<0.05 indicated statistical significance unless specified otherwise.

**REFERENCE**

1. Li X, Bu F, Ma S, Cananzi F, Zhao Y, Xiao M, Min L, Luo C. The Janus-faced role of TRPM2-S in retroperitoneal liposarcoma via increasing ROS levels. *Cell Commun Signal*. 2022 Aug 25;20(1):128.
2. Zhao Y, Yu Y, Li H, Zhang Z, Guo S, Zhu S, Guo Q, Li P, Min L, Zhang S. FAM175B promotes apoptosis by inhibiting ATF4 ubiquitination in esophageal squamous cell carcinoma. *Mol Oncol*. 2019 May;13(5):1150-1165.
